# Sleep duration, timing, and regularity during school, training, and holiday periods in male adolescent soccer players

**DOI:** 10.1038/s41598-026-51685-7

**Published:** 2026-05-07

**Authors:** João Barreira, Elisa A. Marques, João Brito, Fábio Y. Nakamura, Ricardo Pimenta, Pedro Figueiredo

**Affiliations:** 1 Research Center in Sports Sciences, Health Sciences, and Human Development (CIDESD), University of Maia, Maia, 4475-690 Portugal; 2 College of Sport Science, University of Kalba, Kalba, United Arab Emirates; 3 FPF Academy, Portuguese Football Federation, Oeiras, Portugal; 4 CIPER, Faculdade de Motricidade Humana, Universidade de Lisboa, Lisboa, Portugal; 5 Graduate Program in Physical Education, Federal University of Pernambuco, Recife, Brazil; 6 FSI Lab, Football Science Institute, Granada, Spain; 7 Department of Rehabilitation and Optimization of Performance (DROP), Futebol Clube Famalicão - Futebol SAD, Famalicão, Portugal; 8 Research Center of the Polytechnic Institute of Maia (N2i), Maia Polytechnic Institute (IPMAIA), Castêlo da Maia, Maia, 4475-690 Portugal; 9 Physical Education Department, College of Education, United Arab Emirates University, Al Ain, United Arab Emirates

**Keywords:** Youth, Health, Wellbeing, Sports, Monitoring, Health care, Neuroscience, Physiology, Psychology, Psychology

## Abstract

Sleep is essential for adequate physical recovery and performance, but adolescent athletes face unique challenges that can disrupt sleep. Thus, this observational study aimed to monitor sleep in highly trained male adolescent soccer players between three distinct periods: (1) concurrent school and training; (2) morning training only (during the school winter break); and (3) a full holiday period (with no school or training) and assess the influence of morning alarm use on the differences between periods. Thirty-four male academy soccer players (16.6 ± 0.9 years) were monitored for five weeks using wrist-worn actigraphy and daily sleep diaries. Sleep outcomes included time in bed (TIB), total sleep time (TST), sleep timing, and the Sleep Regularity Index (SRI). Linear mixed-effects models were used to compare sleep variables across periods and to test interactions with morning alarm use. Mean sleep duration across the monitoring period was 06:47 ± 01:10 h, with none of the participants achieving the recommended minimum of 8 h of daily sleep. TIB and TST were slightly lower during the training-only period compared with the other periods (− 18 to − 31 min; trivial to small effects). Holidays were characterized by substantially later bedtimes (~ 1.5–2 h; moderate effects) and wake-up times (~ 2 h; moderate effects), without a meaningful increase in sleep duration (3 to 21 min; trivial effects). Marked differences in SRI were observed between periods (moderate to large effects), with higher SRI during structured periods and the lowest regularity during holidays (moderate to large effects). Not using an alarm resulted in longer TIB (+ 87 min; large effects) and TST (+ 66 min; moderate to large effects), particularly during holidays. The main results highlight that highly trained male adolescent soccer players had later sleep timing during holidays, which did not necessarily promote longer sleep; instead, it was associated with reduced sleep regularity. Moreover, morning alarm use negatively influenced sleep opportunity.

**Supplementary Information:**

The online version contains supplementary material available at 10.1038/s41598-026-51685-7.

## Introduction

Adequate sleep is essential for health and daily functioning, and is a major contributor for proper growth and development of children and adolescents^1,2^. Current recommendations suggest that adolescents aged 14–18 years should obtain 8–10 h of sleep per night^3,4^; yet, large-scale studies consistently show that most adolescents sleep less than 7 hours^5^. Adolescent athletes might be even more vulnerable, with studies reporting an average of 6–7 h of sleep per night^6–8^. This inadequate sleep has been shown to negatively impact various features, such as academic performance, mental, cardiometabolic, and emotional health, cognitive performance, mood, and injury risk^9^.

A central reason for short sleep during adolescence is the misalignment between biological and social sleep schedules^10^. Delays in circadian rhythms, combined with slower homeostatic sleep pressure accumulation, naturally shift adolescents’ sleep phase later at night^11^. However, early morning school or training commitments require waking earlier than biologically preferred, contributing to chronic partial sleep deprivation and the accumulation of sleep debt. This debt is typically compensated through extended weekend sleep, a phenomenon known as social jetlag^12^. However, for adolescent athletes, opportunities for catch-up sleep during weekends may be further limited by early morning training sessions or competitions^6^. In addition, adolescents participating in competitive sports may have additional stressors due to conflicting commitments and anxiety related to training and competition^13^. Increased stress and anxiety have been linked to compromised sleep quality and quantity^14^.

Accordingly, periods with less constrained daily schedules, such as school holidays, may represent a valuable opportunity for adolescent athletes to extend their sleep duration. Studies among non-athletic adolescents have shown longer sleep duration during holidays than during school periods^15^. However, research with adolescent athletes is inconsistent: one study reported longer sleep duration during the holidays in adolescent male soccer players^16^, while another study did not find any differences between these periods (school versus holidays) in male and female adolescent athletes^17^. These discrepancies may reflect differences in monitoring methodology (self-reports vs. actigraphy), the duration of the holiday period, type of sport, baseline sleep patterns during the school term, and even sex differences. Given these inconsistencies, additional research using objective measures of sleep is needed.

Importantly, while school holidays can permit increased time in bed and, consequently, longer sleep duration, their unstructured nature may also result in more irregular sleep-wake patterns. During school periods, wake-up times are typically anchored by early morning obligations, helping to maintain regularity. In contrast, unstructured schedules may allow adolescents to follow their naturally later chronotype, potentially increasing night-to-night sleep variability. Sleep regularity may be just as important for psychological well-being as sleep duration, and is an important determinant of mental health, cognitive function, and overall well-being^18^. Greater night-to-night sleep variability has been associated with more stressful life events and increased psychopathology, especially bipolar and depressive symptoms in adolescents^19^. Yet, research on sleep regularity in adolescent athletes remains scarce.

Given the available evidence, the present study aimed to monitor sleep in highly trained male adolescent soccer players during three distinct periods: (1) a period of concurrent school and training; (2) a period of morning training only (during the school winter break); and (3) a full holiday period (with no school or training). The primary objective of the study was to compare actigraphy data across the three periods. A secondary objective was to examine whether wake-up type (alarm vs. no alarm) influenced differences between periods.

## Results

### Participant characteristics

Of the 39 participants who met the school enrollment criterion, 34 (aged 16–18 years; mean age ± SD; 16.6 ± 0.9 years; body mass: 68.0 ± 6.2 kg; height: 176.6 ± 7.0 cm) constituted the final analytical sample based on data quality requirements. Five participants were excluded due to insufficient data in at least two out of the three study periods. Valid sleep data were obtained for all three study periods in most participants; however, 8 participants (24%) provided valid data for only two periods. Among participants with incomplete data, 5 (15%) had insufficient data (i.e., fewer than 5 days) for the concurrent school and training period, 1 (3%) had insufficient data for the training-only period, and 2 (6%) had insufficient data for the holiday period.

### Baseline sleep characteristics

The chronotype of most participants (71%) was intermediate, followed by the morning-type (26%) and, lastly, the evening-type (3%). Based on PSQI and ESS scores, most participants were identified as having good sleep quality and no excessive daytime sleepiness; however, 23.5% did not. Using the ASSQ, most participants (83.3%) were classified as having no sleep difficulties; however, 11 participants (32.4%) had some level of sleep difficulty, ranging from mild (*n* = 8; 23.5%) to severe (*n* = 1; 3%). Eight participants (23.5%) were identified as being at risk of sleep-disordered breathing (loud snoring only = 2, apneas only = 5, both = 1) and 6 participants (18%) were at risk of sleep and performance issues during travel (3 had sleep disturbances only, and 3 had performance issues only).

During the 5-week data collection period, the mean daily values for each sleep parameter across all participants were: time in bed 08:17 ± 01:19 h, total sleep time 06:47 ± 01:11 h, sleep regularity index 72.0 ± 5.2, sleep efficiency 81.9 ± 7.0%, sleep-onset latency 9.9 ± 7.5 min, and wake-after sleep onset 80.4 ± 37.7 min. Average daily bedtime was 00:04 ± 01:36 h, and wake-up time at 08:21 ± 01:54 h. At the individual level, none of the participants obtained an average of 8 h or more of sleep per night during the 5-week period. 5 (15%) participants obtained an average of less than 6 h of sleep per night, 18 participants (53%) slept between 6 and 7 h on average, and 11 participants (32%) slept an average of 7–8 h. Over the 5-week monitoring period, the lowest SRI value was 56 and the highest was 85.

### Sleep comparisons between periods

**Fig. 1:**
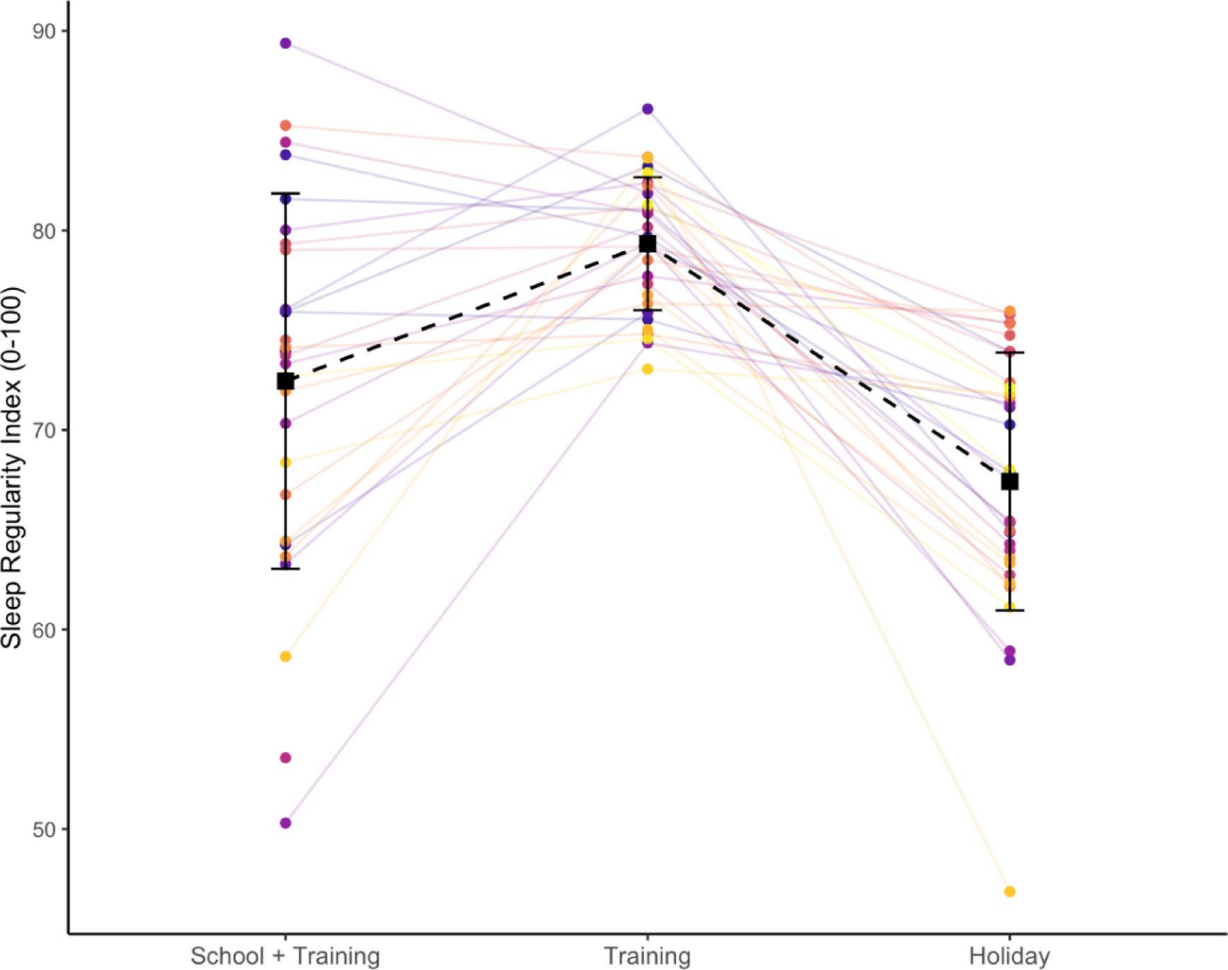
Individual (colored dots and lines) and mean ± SD (black dots and lines) of the sleep regularity index (SRI) during school, training.

The models indicated a significant main effect of period on TIB, TST, and SRI (*p* < 0.01; Table S1), whereas no differences were observed in sleep efficiency. Descriptive comparisons are presented in Table 1. Post-hoc analysis indicated that participants had lower TIB during the training-only period compared with the concurrent school and training period (−24 min, *p* < 0.001, d = 0.14 [95% CI 0.07–0.21]) and with the holiday period (−32 min, *p* < 0.001, d = 0.17 [0.10–0.24]). Consistent with this, results from post-hoc analysis indicated that participants had lower TST during the training-only period compared with the concurrent school and training period (−18 min, *p* = 0.002, d = 0.11 [0.04–0.18]) and to the holiday period (−22 min, *p* < 0.001, d = 0.13 [0.06–0.20]). Finally, during the training-only period, participants had a higher SRI than during the other two periods (concurrent school and training period: +8.0, *p* = 0.001, d = 0.52 [0.25–0.79], and the holiday period: +11.9, *p* < 0.001, d = 0.97 [0.65–1.28]) (Fig. 1). SRI was also higher during the concurrent school and training period compared to holidays (+ 5, *p* = 0.009, d = 0.39 [0.13–0.65]).

**Table 1:** Sleep comparisons between periods.

	Period			*p*
	School + training (*n* = 355)	Training only (*n* = 234)	Holiday (*n* = 247)	
Time in bed (hh:mm)	08:22 ± 01:12	07:58 ± 01:10§	08:29 ± 01:31#	**< 0.001**
Total sleep time (hh:mm)	06:51 ± 01:07	06:33 ± 01:05§	06:54 ± 01:18#	**< 0.001**
Sleep Efficiency (%)	82.0 ± 7.7	82.4 ± 6.6	81.5 ± 6.4	0.386
SOL (min)	9.7 ± 7.2	10.1 ± 7.9	9.9 ± 7.6	0.735
WASO (min)	81.2 ± 40.5	74.3 ± 33.1§	84.9 ± 36.9#	**< 0.001**
Bedtime (hh:mm)	23:22 ± 01:09	23:44 ± 01:11§	01:15 ± 01:47§#	**< 0.001**
Wake-up (hh:mm)	07:45 ± 01:33	07:42 ± 01:32	09:45 ± 01:57§#	**< 0.001**

SOL, sleep onset latency; WASO, wake after sleep onset. n denotes individual observations. § denotes a significant difference from ‘school & training’. # denotes a significant difference from ‘training’. Significant values are in bold.

A significant main effect of period was observed for WASO (*p* < 0.001), bedtime (*p* < 0.001), and wake-up time (*p* < 0.001), while no differences were found for SOL. Post-hoc analysis indicated that WASO was higher during the concurrent school and training period and holiday period compared with the training period (+ 7.6 min, *p* = 0.005, d = 0.11 [0.04–0.18] and + 11.5 min, *p* < 0.001, d = 0.16 [0.09–0.23], respectively). Bedtime was later during the holiday period compared with concurrent school and training period (+ 01:53 h, *p* < 0.001, d = 0.61 [0.53–0.68]) and training period (+ 01:31 h, *p* < 0.001, d = 0.46 [0.39–0.54). Bedtime during the training period was also later compared with the concurrent school and training period (+ 00:22 h, *p* = 0.004, d = 0.11 [0.04–0.18]). Wake-up time was later during the holiday period compared with concurrent school and training period (+ 02:00 h, *p* < 0.001, d = 0.50 [0.42–0.57]) and training period (+ 02:03 h, *p* < 0.001, d = 0.48 [0.41 − 0.56]).

### Secondary analysis: impact of morning alarm use on sleep

Including morning alarm (yes/no) as a moderator improved the marginal and conditional R², particularly for TIB (0.026 and 0.130 vs. 0.199 and 0.309) and sleep duration (0.015 and 0.181 vs. 0.137 and 0.357) regression models. Alarm use was associated with longer time in bed and total sleep time (both *p* < 0.001), with participants who did not use an alarm sleeping approximately 65–87 min longer, on average. The interaction between period and alarm was significant only for time in bed (*p* = 0.009), indicating that the difference between using and not using the alarm was largest during the holidays. No significant main or interaction effects were observed for sleep efficiency (all *p* > 0.05; Table 2).

**Table 2:** Model estimates for the comparisons of sleep metrics between periods.

Predictors	Time in bed			Sleep duration			Sleep efficiency		
	Estimates	CI	*p*	Estimates	CI	*p*	Estimates	CI	*p*
(Intercept)	08:22	08:10–08:33	**< 0.001**	06:50	06:38–07:02	**< 0.001**	81.69	79.83–83.55	**< 0.001**
Period [training only]]	−00:25	−00:37 – −00:13	**< 0.001**	−00:18	−00:28 – −00:07	**0.001**	0.50	−0.35–1.34	0.250
Period [holiday]	00:07	−00:05–00:20	0.251	00:03	−00:07–00:14	0.521	−0.48	−1.33–0.36	0.262
Random effects									
σ^2^	5493.31			4207.24			25.29		
τ_00_	653.10 _ID_			849.25 _ID_			27.91 _ID_		
ICC	0.11			0.17			0.52		
N	34 _ID_			34 _ID_			34 _ID_		
Observations	836			836			836		
Marginal R^2^/conditional R^2^	0.026/0.130			0.015/0.181			0.003/0.526		

Duration results were transformed to hh:mm format for easier interpretation. Significant values are in bold.

## Discussion

The present study compared objective sleep in adolescent male soccer players across three periods: concurrent school and training, training-only, and holiday period. Additionally, the study examined whether the type of awakening influenced these differences. Overall, findings showed consistently short sleep duration across all periods, with small reductions during the training-only period and substantial delays in sleep timing and reduced regularity during the holiday period.

Adolescent athletes have been identified as an at-risk group of sleep disturbances, who may be more likely to experience short sleep duration and reduced sleep quality, compared with young non-athletes^20^. The average sleep duration observed in our study (06:47 h) aligns with previous research in similar populations^6–8^ and reflects the broader trend of adolescents not meeting the recommended 8–10 h per night^21,22^. Biological shifts toward later sleep times, combined with environmental constraints such as school or sports schedules, often reduce sleep opportunity and duration in this population^2,8,20^. As a result, many adolescent athletes likely carry a chronic sleep debt throughout the academic year and sports season^23,24^.

Time in bed and total sleep time per night were slightly lower during the training-only period (−18 to −24 min) compared with the concurrent school and training, and holiday periods, with trivial-to-small effect sizes (d < 0.14). These differences may be attributable to a shift to early-morning training during the training-only period and, despite being small, are consistent with prior evidence that early-morning training sessions reduce sleep opportunity^25,26^. In our study, despite the need to wake-up slightly earlier during the training-only period, participants did not anticipate their bedtimes to compensate for the earlier awakenings. Instead, average bedtimes were even later than during the concurrent school and training period, ultimately reducing sleep opportunity Although the absolute differences were small, these persistent small losses in sleep may accumulate over time, contributing to insufficient sleep throughout periods of intensified early activity. For example, a nightly reduction of approximately 20 min in average sleep results in nearly 2 h of cumulative sleep loss per week.

Consistent with prior research, sleep-wake schedules shifted substantially during the holiday period. Bedtimes were delayed by approximately 1.5–2 h, and wake-up times by roughly 2 h, representing moderate effects. However, in contrast to some findings in non-athletic adolescents^15,27^, the delayed sleep timing observed during holidays did not translate into longer sleep duration. Nonetheless, beyond sleep timings, it is also important to consider behavioral and environmental factors commonly observed during holiday periods. For instance, increased use of electronic devices in the evening, greater participation in social activities, reduced parental or institutional regulation, and more irregular daily routines may all promote later bedtimes and limit sleep extension^27,28^, despite an increased sleep opportunity. Although these variables were not directly assessed in the present study, their potential influence should be acknowledged, and future research should aim to integrate behavioral and contextual measures to better understand sleep patterns during unstructured periods.

The findings also reflect larger bedtime delays offsetting increased morning flexibility in this cohort. Studies with non-athletic adolescents observed smaller changes in bedtimes (~ 01:10 h compared to ~ 01:55 h from school to holidays in our study), but a significant shift in wake-up time (~ 02:20 h compared to 02:00 h from school to holidays in our study), ultimately resulting in increased time in bed^15,27^. Nonetheless, a recent large-scale study conducted with soccer players from various age groups highlighted that adolescent players report needing around 08:30 h of sleep per night^29^, which would place participants of the current study at a sleep deficit of 01:30 h even during holidays. Although sleep timing shifted later during holidays, this did not translate into longer sleep duration.

This study is among the first to examine sleep regularity using the SRI, in adolescent athletes. The main results showed higher SRI values during periods with structured morning obligations, whether it was school or training. The between-period differences were moderate to large (d = 0.52–0.97), representing the strongest effect across all sleep outcomes. The narrow between-subjects variability in SRI during the training-only period (CV = 5%) suggests that even modest external constraints strongly anchor sleep-wake rhythms in adolescent athletes. Despite this, for the duration of data collection, the average SRI in the current soccer players was 72.3, overall lower than data reported in healthy adolescents (SRI: 86.3)^18^ and elite team sport athletes (SRI: 85.1)^30^.

The holiday period was characterized by lower SRI and greater night-to-night variability (CV = 10%), consistent with research showing that unstructured schedules increase irregularity. Prior research has linked irregular sleep with various health concerns, such as physical, mental, and cardiometabolic health, in addition to all-cause mortality^31^. A relationship between low SRI scores and academic performance has also been reported^32^, possibly linked to findings showing greater morning sleepiness and heightened negative mood states in the evening when sleep variability is high^33^. Among student-athletes, a relationship between SRI and sleep onset, offset, cumulative sleep duration, and chronotype was found^26^, indicating that later chronotypes may be associated with more irregular sleep schedules^26^. Our findings show that sleep regularity is highly responsive to environmental structure and may be a useful marker to monitor during youth athletes’ development and promote well-being.

Accordingly, not using an alarm was associated with longer sleep durations (+ 01:06 h) and greater time in bed (+ 01:27 h). The interaction between period and alarm use was significant for time in bed, with the largest difference occurring during the holiday period, when participants likely had the most autonomy over their schedules. While research on morning-alarm use is scarce, our findings align with studies that support that alarm-constrained wake times shorten sleep even on weekends and holidays^33,34^. As such, it may be important for adolescent athletes to have the opportunity to wake up naturally on free days.

The main strengths of this study are the relatively large sample of elite youth athletes and the continuous 5-week actigraphy-based sleep monitoring. However, several limitations should be acknowledged. First, the observational design and reliance on naturally occurring schedule changes limit causal inference. Although we applied thresholds to actigraphy data to improve data quality, an unequal number of observations across periods in unavoidable. Second, school attendance could not be independently verified, and findings may not generalize to female athletes, different sports or differing training structures (i.e., evening training during holidays). Future research should explore sleep regularity and alarm use in more diverse athletic populations and examine how irregular sleep schedules may interact with performance and recovery markers.

In summary, sleep duration remained consistently short across all periods. While increased schedule flexibility during holidays led to later sleep timing, it did not result in meaningful increases in sleep duration and was accompanied by reduced sleep regularity. Alarm use further constrained sleep opportunity, highlighting the importance of considering both schedule structure and wake-up demands when aiming to optimize sleep in adolescent athletes. Considering this, coaches and practitioners may want to be careful when scheduling early morning training during periods of higher schedule freedom, allowing an increase in sleep opportunity.

## Methods

### Study design and setting

This observational study was conducted at a Portuguese soccer academy from 29 of November 2024 to 3 of January 2025. Participants wore actigraphy devices every night to monitor their sleep and kept a sleep diary throughout the study. Additionally, participants completed a set of validated sleep-specific questionnaires prior to the monitoring period.

**Fig. 2:**
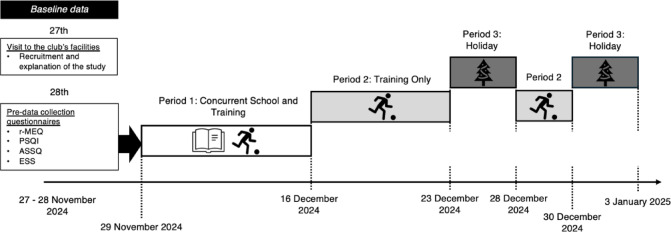
Schematic of the study design.

Prior to data collection, permission from the club and an institutional agreement were obtained. Recruitment and study explanation were conducted on 27 November 2024, and informed consent and baseline questionnaire data collection took place on 28 November 2024. The specific timing of data collection was strategically selected to capture naturally occurring variations in academic and training demands that align with the winter academic calendar, thus providing an ecologically valid design for examining sleep patterns under different lifestyle conditions. The November–January timeframe includes three distinct periods: (1) concurrent school and training period (November–early December), (2) training-only period during school holidays (mid-December), and (3) complete holiday period from both academic and athletic obligations (late December–early January). During this timeframe, objective sleep was continuously monitored using actigraphy devices and sleep diaries, while standardized sleep-related questionnaires were administered at baseline (prior to the objective sleep monitoring period). The longitudinal design enables within-subject comparisons across the three periods, while maintaining ecological validity by using naturally occurring schedule variations. A schematic of the study design is presented in Fig. 2.

All days within each study period were included in the analysis, including free days or rest days (i.e., days without scheduled school and/or training). This approach was adopted to preserve the ecological validity of the study and to capture natural day-to-day variability in sleep patterns. During the concurrent school and training and training-only periods, participants typically had 1 rest day per week without scheduled training, and occasional days without school commitments. The research team was not involved in training planning and load management.

### School and training schedules

During the concurrent school and training period, participants in the U17 team typically attended school in the morning (start time ~ 08:30 h) and engaged in team training sessions at 15:00 h. Participants in the U19 team typically attended school in the afternoon (start time ~ 14:00 h) and engaged in team training sessions at 9:00 h. During the training-only period (school break), training sessions were all scheduled in the early morning for both teams (start time ~ 08:30–09:00 h). During the holiday period, participants had no scheduled school or training commitments.

Participants.

This study represents a secondary analysis of data from a larger observational trial, which will result in several separate publications with distinct research aims. Forty-eight adolescent male highly trained^35^ soccer players from an elite Portuguese soccer club (U17/U19 teams) initially participated in the observational trial (mean ± SD age: 16.7 ± 0.9 years; body mass: 68.7 ± 6.6 kg; height: 176.6 ± 6.6 cm). For this analysis, participants were required to be enrolled in school, resulting in a sample of 39 players. Data inclusion for the final analytical sample followed a two-step approach based on data quality criteria. First, participant data from each period were considered valid for analysis only if they contained at least five days of valid accelerometer sleep data, based on established requirements for reliable sleep parameter estimation^36^. Second, participants were retained in the analytical sample only if they had valid data from at least two of the three study periods (concurrent school and training, training only, holiday), as the primary study objective required within-subject comparisons across different life contexts. Thus, the final analytical sample consisted of 34 participants.

Written informed consent was obtained from participants or their legal guardians (participants under 18 years old) prior to data collection. Pre-specified exclusion criteria included: having travelled across more than two time zones in the past three months, history of major injuries in the previous three months, prior history of a sleep-related disorder, and current medication that may interfere with sleep. None of the participants met any of these criteria. The study was approved by the Ethics Committee of the Portugal Football School (PFS 23/2023).

Measures.

### Sleep-related questionnaires

At study onset, participants completed four online self-administered questionnaires. These included the reduced Morningness–Eveningness Questionnaire (r-MEQ)^37^ for chronotype assessment, the Pittsburgh Sleep Quality Index (PSQI)^38^ for subjective sleep quality, the Epworth Sleepiness Scale (ESS)^39^ for perceived sleepiness, and the Athlete Sleep Screening Questionnaire (ASSQ)^40^ for sleep difficulties and risk of sleep disturbances, all previously validated. All questionnaires were completed prior to the main data collection period.

r-MEQ (Portuguese version^37^: scores on the r-MEQ range from 4 to 25, and participants are classified as morning-type (scores from 18 to 25), neither type (scores from 12 to 17), or evening-type (scores from 4 to 11).

PSQI: 19-item questionnaire organized into seven dimensions: subjective sleep quality, sleep latency, sleep duration, habitual sleep efficiency, sleep disturbances, use of sleeping medication, and daytime dysfunction^38^. A higher score indicates greater sleep disturbances, and the developers have suggested a cut-off score of 5 for the global scale to classify participants with poor sleep quality^38^.

ESS: total daytime sleepiness score ranges from 0 to 24 (0–7 normal, 8–9 mild excessive, 10–15 moderate excessive, and ≥ 16 severe excessive)^39^.

ASSQ: 15-item questionnaire that assesses sleep and circadian factors, including sleep duration, sleep quality, symptoms of insomnia, and chronotype, with a timeframe of “over the recent past”^40^. The sum of items 1,3,4,5,and 6 comprises the sleep difficulty score (SDS), which is categorized into four intensities of the symptoms: none (0–4), mild (5–7), moderate (8–10), and severe (11–17).

### Sleep diaries

Each morning and throughout the study period, participants received a daily link from the research staff and regular reminders from the club staff to maximize compliance. Each diary entry included bedtime, wake-up time, and indication of morning alarm use. A researcher continuously monitored data entries to track compliance and promptly address missing data. Reported bed and wake-up times were compared with automatic sleep scoring from actigraphy. If schedules did not match, the activity graph was analyzed visually, and bed and wake-up times were manually inserted based on the reported values and the visualization.

### Actigraphy sleep monitoring

Sleep was monitored through actigraphy devices (Actigraph LLC wGT3X-BT, Pensacola, USA) worn on the non-dominant wrist. Participants were instructed to use the actigraphy device only at night, placing it on their wrist before going to bed and removing it in the morning after waking up. Device data was analysed using corporate software (ActiLife LLC Pro software v6.13.3, Pensacola, USA). The sampling frequency was set to 50 Hz, and the epoch of activity counts was set to 60 s. Objective sleep measures included time in bed (TIB, total amount of time spent in bed between bed-time and wake-up time), total sleep time (TST, the total amount of sleep obtained), sleep efficiency (percentage of time in bed spent asleep), sleep onset latency (SOL, the amount of time between bedtime and sleep start), wake after sleep onset (WASO, the time spent awake between sleep onset and wake-up time), wake-up time (clock time at which a participant woke at the end of a sleep period), and bedtime (clock time at which participants went to bed to sleep)^41,42^. All sleep variables were determined using *Sadeh’s* algorithm^43^.

Actigraphy data processing

### Actigraphy data processing

The Sleep Regularity Index (SRI) was used as a measure of sleep/wake schedule regularity. The SRI calculates the probability of being in the same sleep/wake state at any given time point 24 h apart and is averaged across the study duration. The index is scaled to a theoretical range between − 100 and 100, but in practice typically ranges from 0 (completely random) to 100 (perfect regularity), using the following formula:

$$\:\mathrm{S}\mathrm{R}\mathrm{I}=-100+\:\frac{200}{\mathrm{M}\left(\mathrm{N}-1\right)}\:{\sum\:}_{\mathrm{j}=1}^{\mathrm{M}}\:{\sum\:}_{\mathrm{i}=1}^{\mathrm{N}}\:{\updelta\:}\left({\mathrm{s}}_{\mathrm{i},\mathrm{j}},{\mathrm{s}}_{\mathrm{i}+1,\mathrm{j}}\right)$$

M = number of daily epochs; N = number of days; *s*_*i.j*_ = 0 for sleep and 1 for wake; and *δ* (*s*_*i.j*_ *≠ s*_*i+1.j*_) = 1 when *s*_*i.j*_ = *s*_*i+1.j*_ *and 0 when s*_*i.j*_ ≠ *s*_*i+1.j*_. SRI was calculated using the sleepreg package for R studio^44^. A binary sleep-wake format was used, with diurnal shifts in the sleep/wake cycle assessed using sleep onset and offset derived from actigraphy devices.

The SRI was calculated for the whole study duration, reflecting sleep regularity throughout the 5-weeks period, and individually for each study period. A minimum of five valid days per period was required for sleep data and SRI calculation, consistent with previous methodological recommendations^44^, but these days were not required to be consecutive. The SRI was calculated using 24-hour-separated epoch pairs, such that only time points with valid corresponding observations exactly 24 h apart contributed to the calculation. Consequently, non-consecutive days could contribute to SRI provided that valid 24-hour pairs were available, whereas gaps without matching data were excluded.

SRI was calculated separately for each study period using all available valid data within that period. For periods that were split into multiple segments (i.e., training-only and holiday periods), all valid data were included, and the calculation inherently accounted for gaps by relying only on valid 24-hour epoch pairs. Each study period was analyzed separately. As such, the final day of a given period did not contribute to any 24-hour pair going forward, extending into the subsequent period.

### Statistical analysis

All descriptive data are presented as mean ± SD. Prior to statistical analysis, normality was checked visually and through the Shapiro–Wilk test, while homogeneity of variances was verified using Levene’s test. Results were considered significant at *p* < 0.05.

Linear mixed models were used to analyze the differences between periods (three levels: concurrent school and training, training only, and holiday period) on the following objective sleep metrics: time in bed, total sleep time, SRI, sleep efficiency, WASO, SOL, bedtime, and wake-up time. Each model included the sleep metric as the dependent variable, period as a fixed effect, and subject ID as a random effect.

For the secondary analysis, the previous linear mixed model analyses were adjusted to include the type of awakening (with or without an alarm) as a moderator. Each model included two fixed effects: period and type of awakening (two levels: yes OR no), and the interaction term.

Models for each sleep variable were compared using the *performance* package^45^. All models were estimated via Restricted Estimated Maximum Likelihood. Model appropriateness was verified using the ‘check_model’ function, checking for normality, linearity, and homogeneity of the residuals. When significance was observed for the main effects, post-hoc pairwise comparison tests using Bonferroni correction were computed to assess differences (i.e., between periods and type of awakening) using the *emmeans* package^46^. Effect sizes (ES) and 95% Confidence Intervals (95% CI) were calculated for the linear mixed model post-hoc analyses by converting the t statistics to d, using the *effectsize* package^47^. ES were calculated using Cohen’s d (d), adjusted for repeated measures, and the magnitude of the ES was interpreted as follows: trivial < 0.2; small > 0.2; moderate > 0.6; large > 1.2; very large > 2.0^48^.

## Supplementary Information

Supplementary Information.

## Data Availability

The datasets used and/or analyzed during the current study are available from the corresponding author upon reasonable request.

## Code availability

The code used in the statistical analysis process of the current study is available from the corresponding author upon reasonable request.
