# Supplementary material for: Sleep duration, timing, and regularity during school, training, and holiday periods in male adolescent soccer players
Source: Sci Rep. 2026 May 7;16:21055. doi: 10.1038/s41598-026-51685-7 (PMC13342076; doi:10.1038/s41598-026-51685-7)
Supplement: Supplementary file 1 — Supplementary Information. [file 41598_2026_51685_MOESM1_ESM.docx]

**Table S1.** Model estimates for the comparisons of sleep metrics between periods.

|  | ***Time in Bed*** | | | ***Sleep Duration*** | | | ***Sleep Efficiency*** | | |
| --- | --- | --- | --- | --- | --- | --- | --- | --- | --- |
| ***Predictors*** | ***Estimates*** | ***CI*** | ***p*** | ***Estimates*** | ***CI*** | ***p*** | ***Estimates*** | ***CI*** | ***p*** |
| (Intercept) | 08:22 | 08:10 – 08:33 | **<0.001** | 06:50 | 06:38 – 07:02 | **<0.001** | 81.69 | 79.83 – 83.55 | **<0.001** |
| Period [Training Only]] | -00:25 | -00:37 – -00:13 | **<0.001** | -00:18 | -00:28 – -00:07 | **0.001** | 0.50 | -0.35 – 1.34 | 0.250 |
| Period [Holiday] | 00:07 | -00:05 – 00:20 | 0.251 | 00:03 | -00:07 – 00:14 | 0.521 | -0.48 | -1.33 – 0.36 | 0.262 |
| **Random Effects** | | | | | | | | | |
| σ^2^ | 5493.31 | | | 4207.24 | | | 25.29 | | |
| τ_00_ | 653.10 _ID_ | | | 849.25 _ID_ | | | 27.91 _ID_ | | |
| ICC | 0.11 | | | 0.17 | | | 0.52 | | |
| N | 34 _ID_ | | | 34 _ID_ | | | 34 _ID_ | | |
| Observations | 836 | | | 836 | | | 836 | | |
| Marginal R^2^ / Conditional R^2^ | 0.026 / 0.130 | | | 0.015 / 0.181 | | | 0.003 / 0.526 | | |

Duration results were transformed to hh:mm format for easier interpretation
